# ONE for All: The Next Step for PLoS

**DOI:** 10.1371/journal.pbio.0040401

**Published:** 2006-11-14

**Authors:** Catriona J MacCallum

## Abstract

*PLoS ONE*, will initiate a radical departure from existing scientific publishing platforms by being more inclusive and by taking advantage of the increasing functionality of internet-based communication.

Most science is not published in *Science*, *Nature*, *Cell*, or even *PLoS Biology*. Indeed, the increasing pressure of submissions, limited page budgets, and the existing reward system by which the value of a paper is placed not on its content but on the venue in which it is published has led most journals to reject a substantial fraction of papers before peer review. The reasons given for rejection are various: the editors may claim that the paper is beyond the scope of a journal, too specialized, of insufficient general interest, or lacking a sufficiently novel advance—even too complicated.

The basis for such decisions is inevitably subjective. The higher-profile science journals are consequently often accused of “lottery reviewing,” a charge now aimed increasingly at the more specialist literature as well. Even after review, papers that are technically sound are often rejected on the basis of lack of novelty or advance. Only recently, I heard of a paper that had been “scooped” by a publication in *Nature* (but it could equally well have been *Science*). The authors submitted the paper to at least three journals (and received seven reviews in total) before their work was accepted. Yet none of the reviewers took issue with the scientific quality of the paper; indeed, some suggested that it was stronger than the first publication. It was rejected because no high-profile journal can afford to be caught publishing the “alsoran,” despite the strong argument that the second paper, replicating and even going somewhat further than the work of the first, is at least as valuable to the scientific record.

Such stories are all too common. But in just a few weeks, the Public Library of Science will launch a new “journal,” *PLoS ONE* (http://www.plosone.org/), that will initiate a radical departure from the stifling constraints of this existing system. Its aims are not only to provide a more inclusive open-access platform for scientific literature—papers will not be rejected on the basis of such subjective justifications as those invoked above—but to reflect far more closely the way that scientific research is conducted by taking advantage of the increasing functionality and flexibility of internet-based communication. All papers that make a valuable contribution to the scientific literature, that are replicable, that are clearly written, and whose conclusions are supported by the data deserve publication. *PLoS ONE* will provide the means to do that swiftly and efficiently.


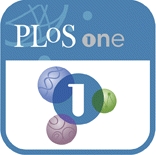


The launch is only the first step—indeed, we refer to this first version of *PLoS ONE* as a beta version to emphasize that it will develop rapidly during the months after launch. Initially, *PLoS ONE* may not look so different from a traditional journal. There is a large and growing editorial board who will handle peer review. Papers, if accepted, will be rapidly posted online (acceptance to publication will be a matter of days) in XML and PDF versions, included in abstracting and indexing services, and they will be deposited in the publicly available archive PubMedCentral. Similar to the other PLoS journals, there will be a publication charge to pay for the cost of review, production, and web hosting (in this case, US$1,250, although there is a discounted price of US$750 for pioneering authors submitting before the official launch). Like the other PLoS journals, the fee will be waived for those without access to appropriate funds. But that is where the similarity ends.

From the start, *PLoS ONE* will be open to papers from all scientific disciplines. Most traditional journals limit the scope of the papers they take and engender artificial barriers between subject areas. As Simon Levin mentioned in his editorial launching the “Challenges” series in *PLoS Biology* [1], no matter the subdiscipline, there are commonalities that unify biology. PLoS strongly agrees, and because such links can be made between all the sciences, *PLoS ONE* is keen to consider submissions not just in biology and medicine but from all of science: physics, chemistry, engineering, computer science, and so on.

Peer review will also be also handled differently. *PLoS ONE* uses a two-stage assessment process starting when a paper is submitted but continuing long after it has been published. Submitted papers will first be scrutinized by an appropriate handling editor from the *PLoS ONE* board who can make a decision to reject or accept a paper (with or without revision) on the basis of their own expertise, or in consultation with other editorial board members or following advice from reviewers in the field. This pre-publication peer review concentrates on objective and technical concerns to determine whether the research has been sufficiently well conceived, well executed, and well described to justify inclusion in the scientific record. If the paper is accepted, the name of the handling editor will be published on the paper as an acknowledgement of their role and responsibility in making the paper publicly available. Such papers may turn out to be citation classics or they may add to data that bear on a question that can only be answered through systematic mining of many papers (such as a meta-analysis)—both types of papers are essential to scientific progress.

But peer review doesn't, and shouldn't, stop there. And this is where the increasing sophistication of web-based tools can begin to play a part. Once a paper is in the public domain post-publication, open peer review will begin. Readers are able to comment on—and rate—articles. Papers will not be a static statement of fact but the beginning of a conversation with the scientific community. Obviously, this will be no free-for-all. Anonymous commenting will not be permitted, and, to take part, commentators will need to conform to the norms of civilized scientific discussion (http://www.plosone.org/ comment_guidelines.php).

The tools that *PLoS ONE* will use to create such web functionality come from a new open-source software project called TOPAZ (http://www.topazproject.org/). *PLoS ONE* will be the first publication to be produced on this platform, and so the *PLoS ONE* and TOPAZ teams are working closely together to meet the growing demand for sophisticated tools and resources to read and use the scientific and medical literature. We are convinced that we will be the first of many publishers, societies, universities, and research communities to take advantage of TOPAZ to produce open-access publications economically and efficiently.

This functionality is just the beginning for *PLoS ONE*. What could now be termed a high-volume, broad-scope online publication will rapidly develop into a much more dynamic platform than can be encompassed by the name “journal.” In science, new connections are often forged between existing subjects, or new fields emerge and become the focus of research interest and funding. Sometimes, these connections grow and persist, but others are more transitory. For example, imagine a situation where you are involved in a project with a particular deadline, such as the 2010 biodiversity targets (http://www.biodiv.org/2010-target/default.aspx), or a large multi-center human-based study, or you may even be organizing a conference or symposium with a specific theme. The papers that arise from such projects can sometimes fall between the narrow scope of existing

All papers that make a valuable contribution to the scientific literature, that are replicable, that are clearly written, and whose conclusions are supported by the data deserve publication.

journals, and researchers have to scan multiple sources to find all of the relevant results. *PLoS ONE* can provide a venue for the review and publication of these papers, which could then be presented online via a single “entry page” or portal.

Moreover, because an open-access model enables each paper to pay for itself, no matter how small the field, the subject can be nurtured. To stimulate this endeavor, *PLoS ONE* will ultimately provide multiple portals as part of its publishing service, where such research can be aggregated for as long as required by a dedicated editorial board regulating the quality and scope of the content displayed. The project might end or the subject might move on, but the papers will still be indexed and listed at *PLoS ONE* and the content made available in perpetuity. Eventually, there is no reason that content from other open-access sources could not be included, including lists of appropriate conferences, blog discussions, debates about the direction that a field is taking, video footage, and so on. The uses of portals will be limited only by their editors' imaginations.

All the journals that PLoS publishes have a specific identity and a particular role. *PLoS Biology* and *PLoS Medicine* are flagship journals whose aims are to help change the culture of scientific publishing, to begin to demonstrate the benefits of open access, and to dispel the myth that great science and important medical advances cannot be published in open-access journals. The PLoS Community Journals, just one year old, also have an important remit. They publish high-quality research catering to particular academic communities. Part of their purpose is to demonstrate that discipline-specific, open-access journals, run by academics within the field (like the majority of journals), can be financially sustainable. *PLoS ONE* is a very different kind of publication that will coexist alongside these journals, increasing our ability to provide venues for authors who want their work published in an open-access forum, and giving us the opportunity to explore all the ways that online publishing can be used to accelerate scientific advance.

We cannot predict what scientific scholarly communication will look like in two years, let alone five, but you can help shape its future. Like the other journals, *PLoS ONE* will only succeed with your help, and we hope you will take an active interest in its development, not only by submitting your own papers but, perhaps more importantly, by contributing to the online discussions and by using the other tools that will be as much a part of *PLoS ONE* as the papers themselves. Even before the launch of *PLoS ONE*, you can be involved by reading and contributing to the blogs that we have started (http://www.plos.org/blogs/). And, in case you were wondering about that excellent but scooped paper I mentioned earlier—it's being published in *PLoS ONE*.
